# Supplementary material for: A computational method for the investigation of multistable systems and its application to genetic switches
Source: BMC Syst Biol. 2016 Dec 7;10:130. doi: 10.1186/s12918-016-0375-z (PMC5142341; doi:10.1186/s12918-016-0375-z)
Supplement: Additional file 1 — Supplementary information. Contains detailed descriptions of the models and algorithms used. (PDF 2350 kb) [file 12918_2016_375_MOESM1_ESM.pdf]

A computational method for the investigation of multi-stable systems and its application to genetic switches.

Miriam Leon, Mae Woods, Alex J. H. Fedorec and Chris P. Barnes

SUPPLEMENTARY INFORMATION

1 Biochemical kinetic models

In this section we provide the priors and equations used for the models given in the main text.

1.1 Gardner switch model priors

The priors used for the analysis of the Gardner switches using StabilityFinder are given below.

Table S1: Gardner switch priors in the deterministic and stochastic cases

Parameters				Species	
a_1	β	a_2	γ	s_1	s_2
0-60	0-5	0-60	0-5	0-100	0-100

1.2 Lu switch models priors

Table S2: Priors of the classical(LU-CS), single positive (LU-SP) and double positive (LU-DP) Lu models.

Parameter	Symbol	CS	SP	DP
Production rate	gx	30-50	1-2	1-100
	gy	30-50	20-25	1-100
Degradation rate	kx	0-0.5	50-55	0-1
	ky	0-0.5	48-52	0-1
Hill coefficient	nxy	1-5	30-35	0-10
	nyx	1-5	0.1-0.2	0-10
Hill thresholds concentration	xxy	100-300	2-3	100-1000
	xyx	100-300	0.4-0.6	100-1000
Transcription rate fold change	lxy	0-0.5	0.02-0.04	0-1
	lyx	0-0.5	0.02-0.04	0-0.2
Hill coefficient	nXX	-	25-30	0-10
	nYY	-	0.01-0.02	0-10
Hill thresholds concentration	xXX	-	0.4-0.5	50-500
	xYY	-	1-3	50-500
Transcription rate fold change	lXX	-	65-72	1-20
	lYY	-	0.02-0.04	1-20

1.3 CS-MA

The classical toggle switch (CS-MA), under the assumption of mass action kinetics, is given by the following reactions:

$$\text{gA} \xrightarrow{\text{geA}} \text{gA} + \text{A}$$
$$\text{gB} \xrightarrow{\text{geB}} \text{gB} + \text{B}$$
$$\text{A} + \text{A} \xrightarrow{\text{dim}} \text{A2}$$
$$\text{A2} \xrightarrow{\text{dim r}} \text{A} + \text{A}$$
$$\text{B} + \text{B} \xrightarrow{\text{dim}} \text{B2}$$
$$\text{B2} \xrightarrow{\text{dim\_r}} \text{B} + \text{B}$$
$$\text{gA} + \text{B2} \xrightarrow{\text{repA}} \text{B2gA}$$
$$\text{B2gA} \xrightarrow{\text{rep\_r}} \text{B} + \text{gA}$$
$$\text{gB} + \text{A2} \xrightarrow{\text{repB}} \text{A2gB}$$
$$\text{A2gB} \xrightarrow{\text{rep\_r}} \text{A2} + \text{gB}$$
$$\text{A} \xrightarrow{\text{deg}} \emptyset$$
$$\text{B} \xrightarrow{\text{deg}} \emptyset$$
$$\text{A2} \xrightarrow{\text{deg\_dim}} \emptyset$$
$$\text{B2} \xrightarrow{\text{deg\_dim}} \emptyset$$

The CS-MA switch was simulated using stochastic dynamics. The stoichimetry matrix and hazards defining the model are shown below:

Table S3: CS-MA stoichiometry matrix

1.0	0.0	0.0	0.0	0.0	0.0	0.0	0.0
0.0	0.0	1.0	0.0	0.0	0.0	0.0	0.0
-2.0	0.0	0.0	0.0	1.0	0.0	0.0	0.0
0.0	0.0	-2.0	0.0	0.0	1.0	0.0	0.0
0.0	0.0	2.0	0.0	0.0	-1.0	0.0	0.0
2.0	0.0	0.0	0.0	-1.0	0.0	0.0	0.0
0.0	-1.0	0.0	0.0	0.0	-1.0	0.0	1.0
0.0	1.0	0.0	0.0	0.0	1.0	0.0	-1.0
0.0	0.0	0.0	-1.0	-1.0	0.0	1.0	0.0
0.0	0.0	0.0	1.0	1.0	0.0	-1.0	0.0
-1.0	0.0	0.0	0.0	0.0	0.0	0.0	0.0
0.0	0.0	-1.0	0.0	0.0	0.0	0.0	0.0
0.0	0.0	0.0	0.0	-1.0	0.0	0.0	0.0
0.0	0.0	0.0	0.0	0.0	-1.0	0.0	0.0

$$
\begin{aligned}
h[1] &= geA \times gA \\
h[2] &= geB \times gB \\
h[3] &= dim \times A^2 \\
h[4] &= dim \times B^2 \\
h[5] &= dim\_r \times B2 \\
h[6] &= dim\_r \times A2 \\
h[7] &= geA \times gA \times B2 \\
h[8] &= rep\_r \times B2gA \\
h[9] &= repB \times gB \times A2 \\
h[10] &= rep\_r \times A2gB \\
h[11] &= deg \times A \\
h[12] &= deg \times B \\
h[13] &= deg\_dim \times A2 \\
h[14] &= deg\_dim \times B2
\end{aligned}
$$

1.4 DP-MA

The DP-MA switch model has the following additional reactions:

$$
\begin{aligned}
\mathrm{A2} + \mathrm{gA} &\xrightarrow{\text{aut 1}} \mathrm{A2gA} \\
\mathrm{A2gA} &\xrightarrow{\text{aut 2}} \mathrm{A} + \mathrm{A2gA} \\
\mathrm{A2gA} &\xrightarrow{\text{aut 3}} \mathrm{A2} + \mathrm{gA} \\
\mathrm{B2} + \mathrm{gB} &\xrightarrow{\text{aut 1}} \mathrm{B2gB} \\
\mathrm{B2gB} &\xrightarrow{\text{aut 2}} \mathrm{B} + \mathrm{B2gB} \\
\mathrm{B2gB} &\xrightarrow{\text{aut 3}} \mathrm{B2} + \mathrm{gB}
\end{aligned}
$$

The stoichiometry matrix and hazards defining the DP-MA model are given below.

Table S4: DP-MA stoichiometry matrix

1.0	0.0	0.0	0.0	0.0	0.0	0.0	0.0	0.0	0.0
0.0	0.0	1.0	0.0	0.0	0.0	0.0	0.0	0.0	0.0
-2.0	0.0	0.0	0.0	1.0	0.0	0.0	0.0	0.0	0.0
0.0	0.0	-2.0	0.0	0.0	1.0	0.0	0.0	0.0	0.0
0.0	0.0	2.0	0.0	0.0	-1.0	0.0	0.0	0.0	0.0
2.0	0.0	0.0	0.0	-1.0	0.0	0.0	0.0	0.0	0.0
0.0	-1.0	0.0	0.0	0.0	-1.0	1.0	0.0	0.0	0.0
0.0	1.0	0.0	0.0	0.0	1.0	-1.0	0.0	0.0	0.0
0.0	0.0	0.0	-1.0	-1.0	0.0	0.0	1.0	0.0	0.0
0.0	0.0	0.0	1.0	1.0	0.0	0.0	-1.0	0.0	0.0
-1.0	0.0	0.0	0.0	0.0	0.0	0.0	0.0	0.0	0.0
0.0	0.0	-1.0	0.0	0.0	0.0	0.0	0.0	0.0	0.0
0.0	0.0	0.0	-1.0	0.0	-1.0	0.0	0.0	1.0	0.0
0.0	0.0	1.0	0.0	0.0	0.0	0.0	0.0	0.0	0.0
0.0	0.0	0.0	1.0	0.0	1.0	0.0	0.0	-1.0	0.0
0.0	-1.0	0.0	0.0	-1.0	0.0	0.0	0.0	0.0	1.0
1.0	0.0	0.0	0.0	0.0	0.0	0.0	0.0	0.0	0.0
0.0	1.0	0.0	0.0	1.0	0.0	0.0	0.0	0.0	-1.0
0.0	0.0	0.0	0.0	-1.0	0.0	0.0	0.0	0.0	0.0
0.0	0.0	0.0	0.0	0.0	-1.0	0.0	0.0	0.0	0.0

$$h[1] = repA \times gA$$
$$h[2] = repB \times gB$$
$$h[3] = dim \times A^2$$
$$h[4] = dim\_r \times B^2$$
$$h[5] = dim\_r \times B2$$
$$h[6] = deg \times A2$$
$$h[7] = rep\_r \times gA \times B2$$
$$h[8] = rep\_r \times B2gA$$
$$h[9] = repB \times gB \times A2$$
$$h[10] = dim \times A2gB$$
$$h[11] = deg \times A$$
$$h[12] = deg \times B$$
$$h[13] = aut\_1 \times B2 \times gB$$
$$h[14] = aut\_2 \times B2gB$$
$$h[15] = aut\_3 \times B2gB$$
$$h[16] = aut\_1 \times A2 \times gA$$
$$h[17] = aut\_2 \times A2gA$$
$$h[18] = aut\_3 \times A2gA$$
$$h[19] = deg\_dim \times A2$$
$$h[20] = deg\_dim \times B2$$

The priors used for the mass action switches are given below.

Table S5: The priors used in the simple (CS-MA) and double positive (DP-MA) mass action models

Parameter	Symbol	Models			
		CS-MA		DP-MA	
		Bistable	Tristable	Bistable	Tristable
Gene expression	geA, B	0 - 10	0 - 10	0 - 10	0 - 10
Dimerization	dim	7 - 15	0 - 3	7 - 15	0 - 3
Monomerization	dim_r	0 - 10	0 - 10	0 - 10	0 - 10
Repression	repA, B	0 - 10	0 - 10	0 - 10	0 - 10
Dissociation of represson	rep_r	0 - 10	0 - 10	0 - 10	0 - 10
Monomer degradation	deg	0 - 10	0 - 10	0 - 10	0 - 10
Dimer degradation	deg_dim	0 - 1	0 - 1	0 - 1	0 - 1
Dimer promoter self-association	aut_1	-	-	0 - 10	0 - 10
Dimer promoter self-activation	aut_2	-	-	0 - 10	0 - 10
Dimer promoter self-dissociation	aut_3	-	-	0 - 10	0 - 10

1.5 Three node switch priors

Table S6: Priors used in the three-node switch

Parameter	Symbol	Range
Production rate	gx	3-5
Degradation rate	kx	0-0.2
Hill coefficient	nxy	0-2
Hill thresholds concentration	xxy	140-160
Transcription rate fold change	lxy	0-0.2
Hill coefficient	nxx	2-4
Hill thresholds concentration	xxx	90-110
Transcription rate fold change	lxx	8-12

1.6 LU-DP posterior distribution

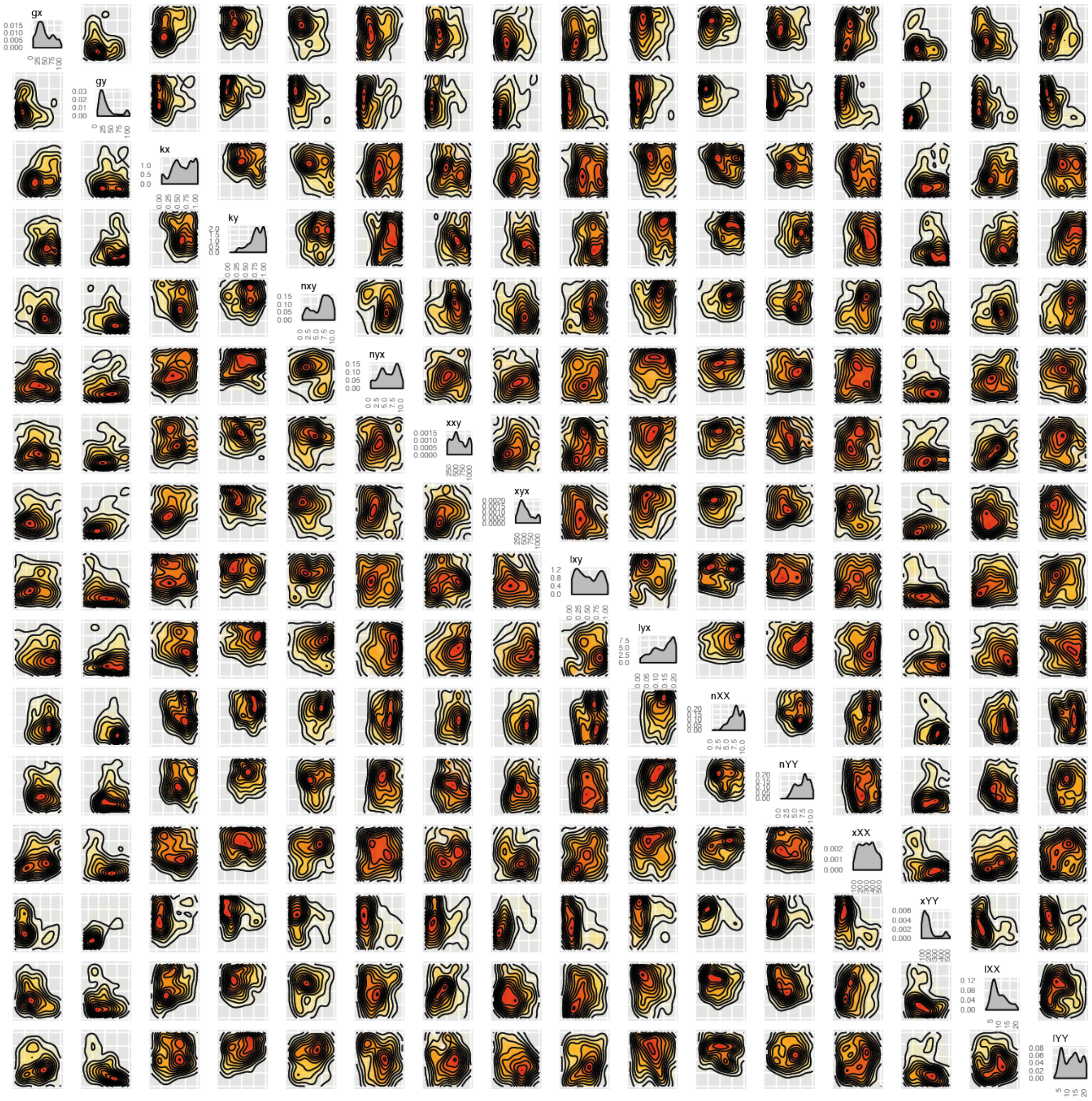

Figure S1: The LU-DP posterior distribution

1.7 Deterministic Gardner model basins of attraction

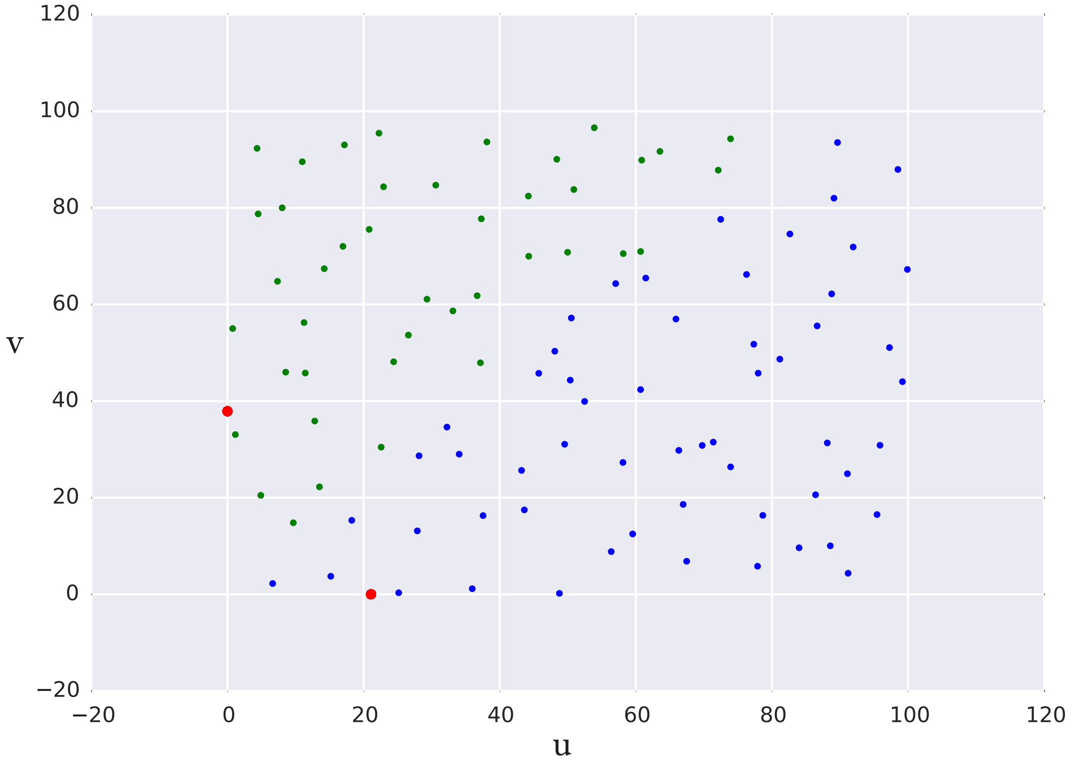

Figure S2: Basins of attraction of the deterministic Gardner model. The red points represent the steady state values. The green and blue points represent the initial conditions resulting to each steady state.

2 Clustering methods

2.1 Deterministic models

Algorithm 1 Clustering the steady state deterministic simulation results

```
for each data point do
    if first point then
        Make first cluster
        cluster counter = 1
    else
        for each cluster do
            if cluster within cluster means ± delta then
                Add to existing cluster
                Update means of clusters
            end if
            if reached_end and not assigned to cluster then
                cluster counter += 1
                Add new cluster
            end if
        end for
    end if
end for
```

2.2 Stochastic models

2.2.1 Gap statistic

Algorithm 2 Choosing the optimal number of clusters

```
function WK(clusters, cluster_centres)
    for each cluster do
        for each point in cluster do
            a = matrix norm (cluster_centre − point)
        end for
        dk = ∑((a)^2) × (2× number of points in cluster)
    end for
    wk = ∑(dk) / (2×(numberofpointsincluster))
    return wk
end function

function GAP_STATISTIC(data, cutoff)
    ks = [1,2,3,4]
    for k in ks do
        cluster_centres, clusters = KMEANS(data, k, cutoff)
        Wk = log(WK(clusters, cluster_centres))
        Create references datasets
        for each references dataset do
            cluster_centres, clusters = KMEANS(data, k, cutoff)
            BWk = log(WK(clusters, cluster_centres))
        end for
        Wkb = ∑(BWk) / 10
        sk = sqrt(∑((BWk−Wkb)^2 / 10))
    end for
    sk = sk × sqrt(1 + 1/B)
    return ks, Wk, Wkb, sk, data_centres, clusters
end function

function DISTANCE(data, cutoff)
    ks, logWks, logWkbs, sk, clusters_means, clusts = GAP_STATISTIC(data, cutoff)
    gaps = logWks − logWkbs
    optimum number of clusters = gaps[i] ≥ (gaps[i+1] − sk[i+1])
    return cluster_counter, clusters_means
end function
```

2.2.2 K-means clustering

Algorithm 3 Clustering stochastic case

```
function KMEANS CLUSTERING(data, k, cutoff)

    function UPDATE_CENTRES(old_centres, values)
        centre_coords = mean for each dimension
        shift = GETDISTANCE(centre_coords, old_centres)
        return shift, centre_coords
    end function

    function GETDISTANCE(a, b)
        dist = √((a[x] − b[x])² + (a[y] − b[y])²)
        return dist
    end function

    while True do
        for each point in data do
            for each cluster do
                dist = GETDISTANCE(point, cluster centre)
            end for
            Find cluster with minimum distance
            Repopulate clusters
        end for
        biggest_shift ← 0
        for as many times as there are clusters do
            shift, cluster centres = UPDATE_CENTRES(old_centres, clusters)
            biggest_shift = max between shift, biggest_shift
        end for
        if biggest_shift ≤ cutoff then
            break
        end if
    end while
    return cluster_centres, clusters
end function
```

3 Effect of variance on Gardner model posterior

The posterior of the deterministic model shown in Figure2C in the main text, parameters a_1 and a_2 have an upper limit of values they can take and still create a bistable switch. In order to test this result, we find the roots of the system for large values of a_1 and a_2 in order to see if three roots are still found, two stable and one unstable. The results as shown in Figure S3 indicate that the system is still bistable for increasing values of a_1 and a_2. This suggests that the upper limit found with StabilityFinder is an artefact of the variance limit imposed on the system. In order to find the steady states we impose an accepted distance from a given total variance for each model. When the two clusters of steady state values are too far apart, this increases total variance of the system and would consequently be rejected in StabilityFinder.

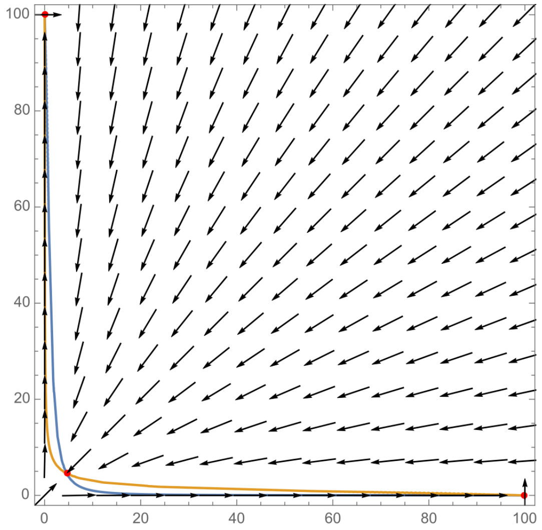

Figure S3: Solving the Gardner toggle switch. The parameters values used are: a_1, $a_2 = 100$ and $\beta, \gamma = 2$. The system has three roots, of which one was found to be unstable and the other two stable. This result disagrees with that found in StabilityFinder, that a_1 and a_2 have an upper limit of 30.
